# Probing the Cr^3+^ luminescence sensitization in *β*-Ga_2_O_3_ with ion-beam-induced luminescence and thermoluminescence

**DOI:** 10.1038/s41598-023-31824-0

**Published:** 2023-03-25

**Authors:** D. M. Esteves, A. L. Rodrigues, L. C. Alves, E. Alves, M. I. Dias, Z. Jia, W. Mu, K. Lorenz, M. Peres

**Affiliations:** 1 INESC MN, Rua Alves Redol 9, 1000-029 Lisbon, Portugal; 2 IPFN, Instituto Superior Técnico, University of Lisbon, Av. Rovisco Pais 1, 1049-001 Lisbon, Portugal; 3 C2TN, Instituto Superior Técnico, University of Lisbon, Estrada Nacional 10, km 139.7, 2695-066 Bobadela, Portugal; 4 DECN, Instituto Superior Técnico, University of Lisbon, Estrada Nacional 10, km 139.7, 2695-066 Bobadela, Portugal; 5 State Key Laboratory of Crystal Materials, Shandong University, Shandanan Street 27, Jinan, 250100 China

**Keywords:** Electronic properties and materials, Semiconductors, Applied physics

## Abstract

Ion-beam-induced luminescence (IBIL) measurements were performed in Cr-doped *β*-Ga_2_O_3_ using both protons and helium ions, showing a strong enhancement of the Cr^3+^ luminescence upon ion irradiation. Theoretical modelling of the IBIL intensity curves as a function of the fluence allowed estimating the effective cross-sections associated with the defect-induced IBIL enhancement and quenching processes. The results suggest that sensitizing the Cr^3+^ luminescence is more efficient for H^+^ than for He^+^ irradiation. Thermoluminescence (TL) studies were performed in the pristine sample, with no TL signal being observed in the spectral region corresponding to the Cr^3+^ emission. In agreement with the IBIL study, upon ion irradiation (with either protons or helium ions), this TL emission is activated. Moreover, it can be quenched by annealing at 923 K for 10 s, thus revealing the role played by the defects induced by the irradiation. These results show that the irradiation-induced defects play a major role in the activation of the Cr^3+^ luminescence, a fact that can be exploited for radiation sensing and dosimetry.

## Introduction

*β*-Ga_2_O_3_ is an emerging semiconductor with a monoclinic structure which has promising applications due to its properties, such as its high thermal and chemical stability, its wide bandgap of ~ 4.9 eV at room temperature and its large breakdown electric field of ~ 8 MV/cm^1–3^. Some of these applications include optoelectronic devices^4^, high-power electronics^5^, solar-blind ultraviolet (UV) photodetectors^6^ and gas sensors^7^. In the context of optical applications, and due to its high transparency, *β*-Ga_2_O_3_ is a good host material for optically active centers in the spectral region spanning from the UV to the infrared (IR). In particular, in addition to the UV/blue luminescence commonly observed in nominally undoped *β*-Ga_2_O_3_^8^, Cr-doping has been shown to provide an efficient red/near IR (NIR) emission assigned to Cr^3+^ intraionic transitions^9–11^. In this context, Cr-doped *β*-Ga_2_O_3_ shows great potential to be used in ionizing radiation detectors for active and passive optical dosimetry during radiation therapy and diagnosis. In particular, the in vivo applications are especially promising, since the emission lies within the so-called first biological window (spanning from 700 to 950 nm), where biological tissue absorbs the least^12^. Additionally, a red/IR scintillator crystal working as an active dosimeter has advantages, namely the possibility to avoid the noise due to the Cherenkov radiation in the blue/UV wavelengths^13^, as well as the noise due to the blue scintillation of plastic optical fibers used to guide the light from the crystal to the detector^14^.

Many luminescence studies have been performed in *β*-Ga_2_O_3_. Apart from the case of nanowires^15^, no luminescence has been observed in a close vicinity of the band gap energy for *β*-Ga_2_O_3_^16^ (4.9 eV/ ~ 250 nm). Broad bands at lower energies (UV/blue spectral region) have been reported and attributed to optical transitions involving the recombination of self-trapped excitons (STE) and donor–acceptor pair (DAP) and free-to-bound transitions. The emission bands associated with STE recombination are broad due to the lattice distortion and strong coupling to phonons^16^. Additionally, it has also been proposed that these emissions arise due to the tunnel recombination of an electron on a donor (such as a neutral O vacancy, V_O_^×^) with a hole on an acceptor, such as a triply-negatively charged Ga vacancy (V_Ga_′′′) or a charged pair of vacancies (V_O_–V_Ga_)′^8,17^. The ion-beam-induced luminescence (IBIL) associated with these emissions has also been studied, with its integrated intensity decreasing as a function of the fluence due to the creation of defects that act as competing non-radiative recombination centres^18^. There are also several studies concerning the optical properties of the Cr dopant in *β*-Ga_2_O_3_. In particular, at room temperature, the characteristic luminescence associated with Cr in the 3+ oxidation state extends at least from 650 to 850 nm, corresponding to two sharp lines (the R-lines, with R_2_ centered at ~ 690 nm and R_1_ at ~ 697 nm) superimposed on a broad band^9,19^. This luminescence has been previously explored in different applications, ranging from tunable optical microcavities^20^ to thermometers^21^. Regarding the optical activation of the Cr^3+^ ions in *β*-Ga_2_O_3_, the excitation processes are not fully understood yet, due to the large number of possible defect states in this material that can be responsible for the sensitization. Such processes include energy or charge transfer between traps in *β*-Ga_2_O_3_ and Cr^3+^ ions^22–25^. Recent IBIL measurements monitored at the Cr^3+^ emission wavelengths show a strong enhancement of the emission intensity as a function of the irradiation fluence in a conductive sample, thus suggesting that irradiation-induced defects play a major role in the activation of this luminescence^23^. Moreover, several works show a clear dependence of the Cr^3+^ luminescence yield on the electrical conductivity of the sample, with insulating samples showing higher yields than conductive ones^23,26,27^. While this dependence has been suggested to be related with different Cr^2+^/Cr^3+^ concentrations, due to different Fermi level positions inside the bandgap, there is no direct experimental evidence of Cr^2+^ ions in *β*-Ga_2_O_3_^23,27,28^. In fact, recent works found no changes in the Cr^3+^ charge state irrespective of ion irradiation and irrespective of co-doping with Mg, both of which may pin the Fermi level by introducing deep defect levels within the bandgap^23^.

In view of this, there is a plethora of defect levels located within the wide bandgap of *β*-Ga_2_O_3,_ as reported in several theoretical and/or experimental works employing techniques such as deep level transient/optical spectroscopy (DLTS/DLOS) or thermoluminescence (TL)^22,24,29–35^. While the unambiguous assignment of these traps is notoriously difficult, some of these levels have been attributed to intentional or unintentional dopants, such as Fe, Cr and Mg or to intrinsic defects, such as O vacancies, or complex defects involving them. In particular, a defect complex involving Fe and intrinsic defects, with an energy level lying 0.7 eV below the conduction band minimum, was reported, and a correlation between its concentration and the Cr^3+^ luminescence was observed^22^. Additionally, the charge transfer level Fe^2+^/Fe^3+^ (energy level lying 0.78 eV below the conduction band minimum^29^) was proposed to act as a charge transfer channel to Cr ions in the 4+ charge state^35^.

Therefore, while the Cr^3+^ emission has been shown to depend on the electrical conductivity^23,26,27^ and on the presence of certain defect levels within the band gap^22^, the full excitation processes are still unclear. In this context, this paper presents a detailed IBIL study performed on Cr-doped *β*-Ga_2_O_3_ using two different ions (H^+^ and He^+^) in order to assess the evolution of the IBIL intensity associated with the Cr^3+^ ions with the irradiation fluence. This study was complemented with TL measurements to assess the induced defect levels after the irradiation, for each of the ions.

## Results and discussion

A Cr-doped *β*-Ga_2_O_3_ single crystal with (100) surface orientation was grown by a modified edge-defined film-fed growth (EFG) method^36^. Moreover, the sample was observed by particle-induced X-ray emission (PIXE) to contain trace amounts of Fe, which is a common contaminant in the context of the EFG method. Further information on this sample and preliminary IBIL results have been published elsewhere^23,36^.

The IBIL was measured with 600 keV H^+^ and 2000 keV He^+^ in a μ-probe set-up at a 2.5 MV Van de Graaff accelerator^37^. Further details on the experimental setup can be found elsewhere^18,23,38^. The nominal beam currents and irradiation areas used were 400 pA and 4.3 × 10^−3^ cm^2^ for the H^+^ irradiation and 100 pA and 4.0 × 10^−3^ cm^2^ for the He^+^ irradiation. The energies are chosen so as to achieve similar vacancy profiles for the two ions (see the supplementary material), according to *Stopping and Ranges of Ions in Matter* (SRIM) Monte Carlo simulations^39^. Moreover, since the ionization is about four times higher for He^+^ than for H^+^, in order to achieve similar excitation densities, the beam current was chosen four times smaller for He^+^ than for H^+^, with similar irradiation areas. The luminescence was monitored in situ using a Hamamatsu Photonics PMA-12 Photonic multichannel analyzer C10027-01.

The TL measurements were performed using a Thermoluminescence/Optically-stimulated luminescence set-up. The excitation was performed using a ^90^Sr/^90^Y *β*^*−*^ source and each irradiation had a duration of 400 s. The measurement was performed with a heating rate of 2 K/s. A commercial detection filter RG630 was employed in order to monitor exclusively the spectral region with wavelengths above (630 ± 6) nm^40^, where the Cr^3+^ emission lies. Moreover, the employed photomultiplier tube (PMT) has an upper detection limit of 630 nm, so that a very narrow wavelength window is being assessed by these measurements.

**Figure 1:**
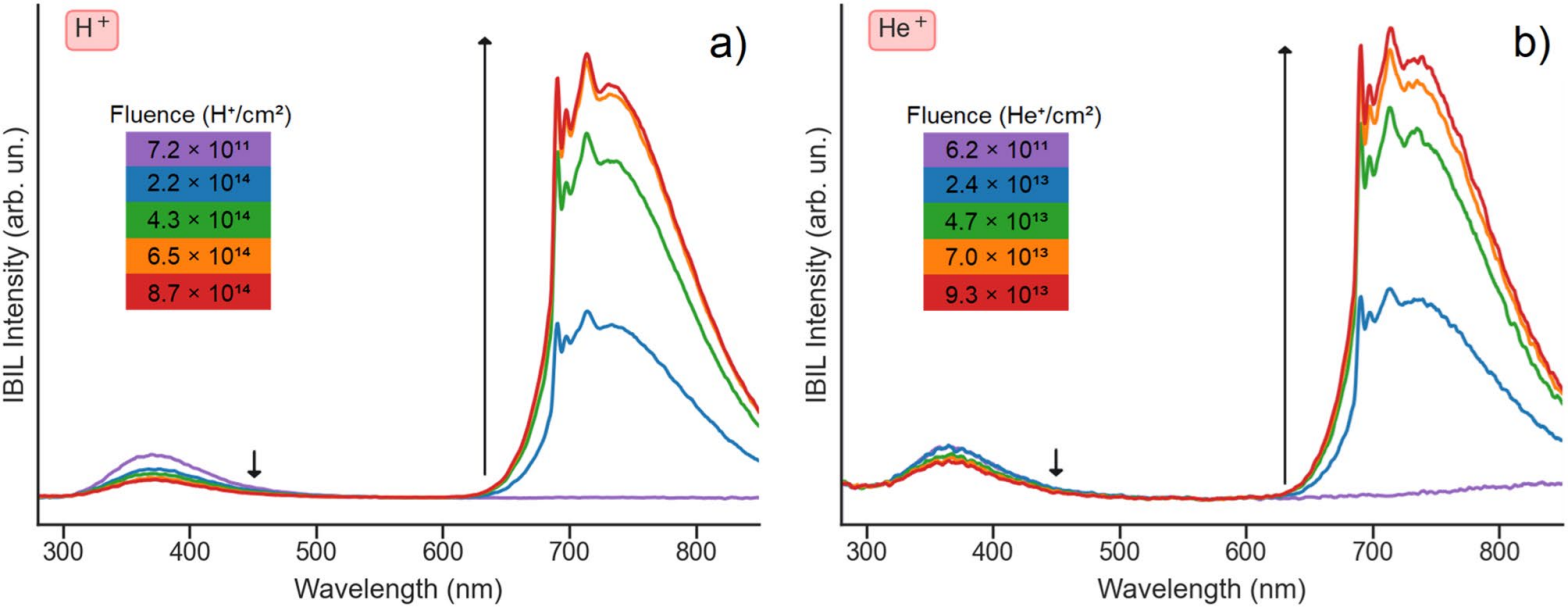
Evolution of the IBIL spectra with increasing fluences, upon irradiation with either H^+^ ions (**a**) or He^+^ ions (**b**). The arrows indicate the tendency of the intensity with increasing fluence.

The IBIL spectra (Fig. 1) are observed to undergo major changes as the irradiation progresses for both ion species. In particular, the Cr^3+^ characteristic red/IR luminescence, negligible in the first spectra (shown in purple in Fig. 1a,b), is subsequently enhanced, while the UV/blue band is quenched as the irradiation fluence increases. Moreover, the overall shape and position of the emission bands is not altered during the irradiation and is similar for the two ion species. Since the enhancement does not depend directly on the ion species, this suggests that the sensitization mechanism is related with the defects induced by the ions in the material.

In order to better understand the very complex dynamics of defect creation, two models were employed. On the one hand, following M. Peres et al*.*^18^, the quenching of the UV/blue emission can be described by the model by P. Sullivan and R. A. Baragiola^41^. This model provides a simple expression compatible with the typically observed decay of the IBIL intensity, by assuming that the induced damage is proportional to the energy deposited in the sample via the nuclear interaction, whereas the luminescence is proportional to the energy deposited via the electronic interaction. The latter leads to the excitation of electrons into the conduction band or to defect states within the bandgap. These inelastic excitations can then relax either by non-radiative processes or produce luminescence upon the electron–hole recombination. Therefore, the integrated IBIL intensity *L* is given as a function of the fluence *F* according to:

1 $$L\left( F \right) = \frac{{L_{0} }}{{1 + k\left( {{\text{e}}^{\sigma F} - 1} \right)}} + c,$$

where *k* is the ratio between the non-radiative and radiative transition rates and *L*_0_ is the luminescence intensity extrapolated to *F* = *0* (zero fluence) and plays the role of a normalization factor for the IBIL intensity for very low fluences (at the beginning of the irradiation). Moreover, *σ* is an effective cross-section for damage formation averaged over the path of the incident ion, as obtained from SRIM simulations by dividing the average number of vacancies created per unit length and per incident ion over the entire irradiated depth (4.30 µm for H^+^ and 4.20 µm for He^+^ ions) by the atomic density of the material^18,42^ (9.45 × 10^22^ atoms/cm^3^, assuming a mass density of 5.88 g/cm^3^)^1,3^. The fluence-independent term *c* accounts for background radiation from sputtered and backscattered particles or from possible indirect processes of excitation taking place far away from the region where the primary nuclear and electronic interactions occur^41^.

On the other hand, the enhancement of the red/IR Cr^3+^ emission is described in this work by an adaptation of the model proposed by C. Manfredotti et al*.*^43^ for diamond, where the pristine sites are converted by irradiation into radiative centers with an effective cross-section *σ*_p→r_ and, in turn, these radiative centers can be converted into non-radiative centers with an effective cross-section *σ*_r→nr_. This model therefore assumes that there are no damage recovery processes (i.e*.*, the created defects cannot be converted back to pristine sites) and moreover assumes that pristine sites can only be converted to radiative centers and not directly to non-radiative centers. Assuming that the IBIL is proportional to the concentration of radiative centers, the integrated IBIL intensity *L* is given as a function of the fluence *F* according to:

2 $$L\left( F \right) = K\frac{{\sigma_{{{\text{p}} \to {\text{r}}}} }}{{\sigma_{{{\text{p}} \to {\text{r}}}} - \sigma_{{{\text{r}} \to {\text{nr}}}} }}\left( {{\text{e}}^{{ - \sigma_{{{\text{r}} \to {\text{nr}}}} F}} - {\text{e}}^{{ - \sigma_{{{\text{p}} \to {\text{r}}}} F}} } \right),$$

where *K* is a proportionality constant.

**Figure 2:**
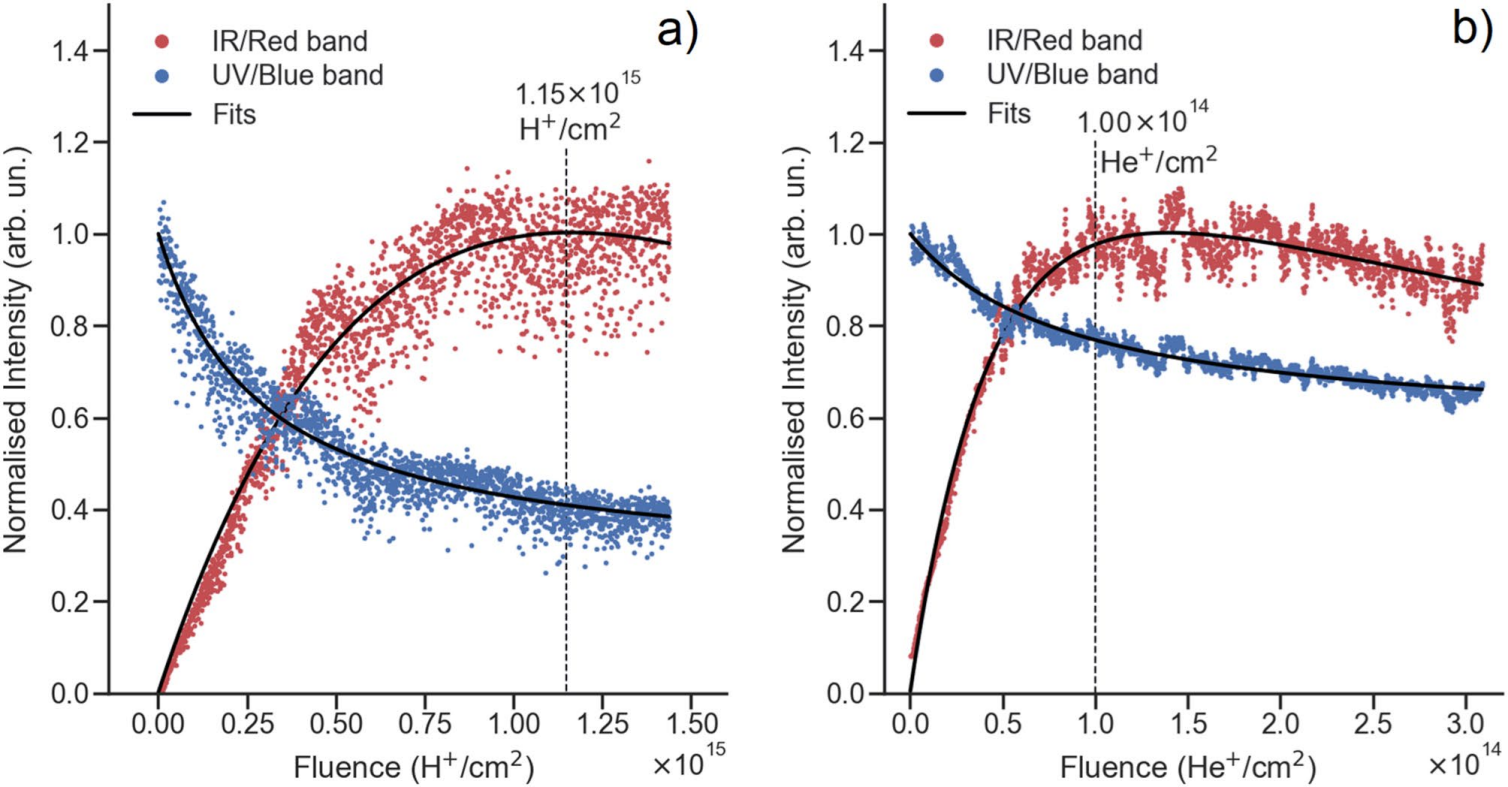
Normalized integrated intensity of the red/IR and UV/blue bands as a function of the H^+^ fluence (**a**) or He^+^ fluence (**b**).

Figure 2 shows the evolution of the normalized integrated IBIL intensity as a function of the irradiation fluence. The least-squares fitting parameters obtained by applying Eqs. (1) and (2) for each ion and luminescence band are shown in Table 1, as well as their associated error.

**Table 1:** Fit parameters of the integrated IBIL intensity for the UV/blue and red/IR bands for the irradiation with 600 keV H^+^ ions or 2000 keV He^+^ ions.

		UV/Blue band parameters			Red/IR band parameters		
Ion	*L*_0_ (arb. un.)	*k*	*σ* (cm^2^)	*c* (arb. un.)	*K* (arb. un.)	*σ*_p→r_ (cm^2^)	*σ*_r→nr_ (cm^2^)
H^+^	0.08 ± 0.01	(6.55 ± 0.08) × 10^3^	5.27 × 10^−19^	0.030 ± 0.001	5.2 ± 1.7	(7.53 ± 2.44) × 10^−16^	(9.77 ± 2.78) × 10^−16^
He^+^	0.25 ± 0.01	(2.21 ± 0.04) × 10^3^	5.13 × 10^−18^	0.317 ± 0.001	141.9 ± 3.1	(9.38 ± 0.18) × 10^−16^	(2.41 ± 0.02) × 10^−14^

The results show that the Cr^3+^ luminescence strictly increases up to a saturation point, while the UV/blue emission, characteristic of nominally undoped *β*-Ga_2_O_3_, strictly decreases due to the creation of defects that act as non-radiative recombination centers^18^ and due to the increasing radiative recombination via Cr^3+^ ions. Moreover, Eqs. (1) and (2) are observed to provide an accurate description of the experimental points, with the dispersion of the intensities being mainly due to fluctuations of the beam current.

Regarding the fitting parameters associated with the UV/blue emission, obtained using Eq. (1), it is possible to observe that the values of the ratio of non-radiative to radiative transition rates (*k*) are of the same order of magnitude for both H^+^ and He^+^ ions. Moreover, this order of magnitude is similar to those previously reported in the literature in *β*-Ga_2_O_3_ flakes and Mn-doped ZnGa_2_O_4_ fibers^18,42^; as also pointed out in the literature, the considered cross-section *σ* only takes the creation of vacancies into account, thus ignoring other luminescence-activation mechanisms, such as the activation of electron traps due to ionization processes. Therefore, the value of *σ* may be underestimated, which leads to an overestimation of *k*.

**Figure 3:**
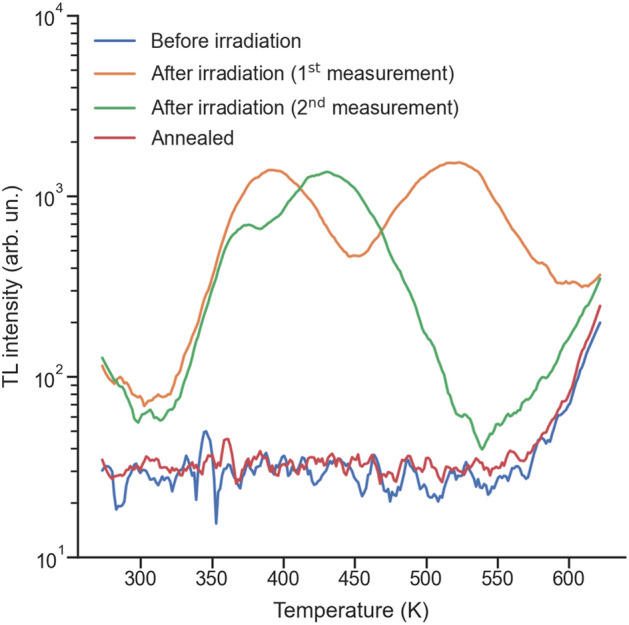
TL glow curves (in logarithmic scale), obtained before and after H^+^ irradiation (showing the first two consecutive measurements) and after annealing in situ at 923 K for 10 s. These glow curves were not corrected for the background.

Regarding the fitting parameters associated with the Cr^3+^ emission, obtained using Eq. (2), the cross-section for the conversion of radiative to non-radiative centers is almost 2 orders of magnitude larger in the case of He^+^ than in the case of H^+^, in agreement with the larger value for the saturation fluence in the case of H^+^, but higher than expected from the SRIM simulations that predicted a scaling factor of 11.5. Unexpectedly, the obtained cross-sections for the conversion of pristine sites into radiative centers are of the same order of magnitude (~ 10^−16^ cm^2^) for both ions. This fact suggests that the nature of defects is different for each ion species, leading to a less efficient sensitization of the Cr^3+^ emission for the He^+^ ions. In fact, since He^+^ ions are heavier, the collision cascades they induce are denser than the ones induced by the H^+^ ions, thus possibly giving rise to different types of defects, such as di-vacancies or larger clusters of defects. This may explain the less efficient sensitizing of the Cr^3+^ emission, while increasing the quenching at higher fluences. The impact of the density of the collision cascades in the disorder and strain accumulation in *β*-Ga_2_O_3_ has recently been demonstrated in the literature, which presents interesting opportunities in the context of defect engineering^44^. Finally, it should be noted that, in this context, this model provides only an effective and simplified description of the very complicated processes of defect accumulation in the sample, which depends on the nature of the defects and the detailed physical mechanisms leading to the sensitization of the Cr^3+^ ions. Some of the mechanisms previously suggested in the literature involve the transfer of either energy or charge between trap states within the band gap and the Cr^3+^ ions, but the true excitation processes are notoriously difficult to pinpoint^22–25^. In this context, TL is an invaluable technique in order to assess the creation of electron traps within the band gap, and can thus be used to complement the IBIL study above. The TL experimental procedure was performed in four steps:

- First, TL measurements were performed in a pristine sample, in one single temperature sweep starting from room temperature up to about ~ 620 K, with a heating rate of 2 K/s.
- Afterwards, three H^+^ homogeneous irradiations were sequentially performed using three different energies: 1.0, 1.5 and 2.0 MeV. Each irradiation amounted to a fluence of about 1 × 10^15^ cm^−2^. The usage of three energies allows a larger irradiated volume to be achieved, which in turn increases the luminescence signal. According to SRIM simulations^39^, the first ~ 25 μm are damaged by the H^+^ irradiation (see the supplementary material), which correspond to the emitting volume.
- Next, the irradiated sample was measured by TL in two consecutive measurements, which were performed in similar conditions, i.e., with the same temperature range and heating rate.
- Finally, the TL measurement was repeated after the sample was annealed in situ at a temperature of 923 K for 10 s. The glow curves obtained at each of these steps are shown in Fig. 3, where the spectra are not corrected for the background (namely, the dark current of the PMT and the blackbody radiation).

It is possible to observe a clear evolution of the TL glow curves before and after the H^+^ irradiation. In fact, before irradiation, no signal was observed, apart from the blackbody infrared emission of the sample (above ~ 600 K). It should be emphasized that the absence of a TL signal does not mean that the sample does not have any traps; instead, it means either that the luminescence associated with the radiative recombination of electrons released from the traps does not occur at the probed wavelengths (a small window around 630 nm) or that these defects are not active and cannot trap and/or release electrons prior to the irradiation^45,46^. The first measurement after the irradiation shows, at least, two peaks centered at ~ 390 K and ~ 520 K; however, on the second measurement, the curve changes, presenting a peak centered at ~ 430 K with a smaller shoulder at ~ 370 K. This alteration hints at the removal of some defects during the first measurement, in which the sample was heated up to 623 K, which is close to the recovery temperatures of proton-irradiation-induced defects indicated in the literature (~ 650 K)^30^. Hence, this difference in the two glow curves obtained at each measurement can be attributed to the unintentional annealing of the sample during the first measurement.

Nevertheless, according to our previous IBIL study with annealing steps, the Cr^3+^ emission is only completely quenched above ~ 550 °C (~ 823 K)^23^, which agrees with the fact that we are still able to observe TL after the first measurement. Moreover, the curve obtained during the second measurement was reproduced in all the subsequent measurements performed afterwards (not shown), suggesting that the defect states that were not removed during the first temperature sweep are stable within the measuring temperature range. Finally, the annealing step at 923 K completely removed the TL signal associated with the Cr^3+^, which is also in agreement with previous IBIL and DLTS results^23,30^. Moreover, the TL measurements performed on both pristine and completely annealed samples did not show any additional glow peaks, which also excludes the possibility that the electron irradiation can contribute to the measured signal.

After these first measurements, an additional, more detailed study was performed using the *T*_M_–*T*_STOP_ method^47^ and also by computationally deconvoluting the glow curve using first and general kinetic order glow peaks^48^ (see the supplementary material). The estimated activation energies of the traps lie approximately between 0.6 and 0.9 eV below the minimum of the conduction band and can be tentatively assigned to different defects involving O vacancies or Fe contaminants, based on previous TL and DLTS/DLOS works^22,24,30,31,33–35,49^.

There are several possible mechanisms that can explain both the IBIL and TL results. On the one hand, the irradiation-induced defects—either the point defects themselves or complex defects—may act as charge or energy transfer channels to the Cr^3+^ ions. Moreover, the irradiation defects have been previously reported to pin the Fermi level lower in the band gap^30^; this can change the oxidation state of a given defect and thus activate the excitation process.

**Figure 4:**
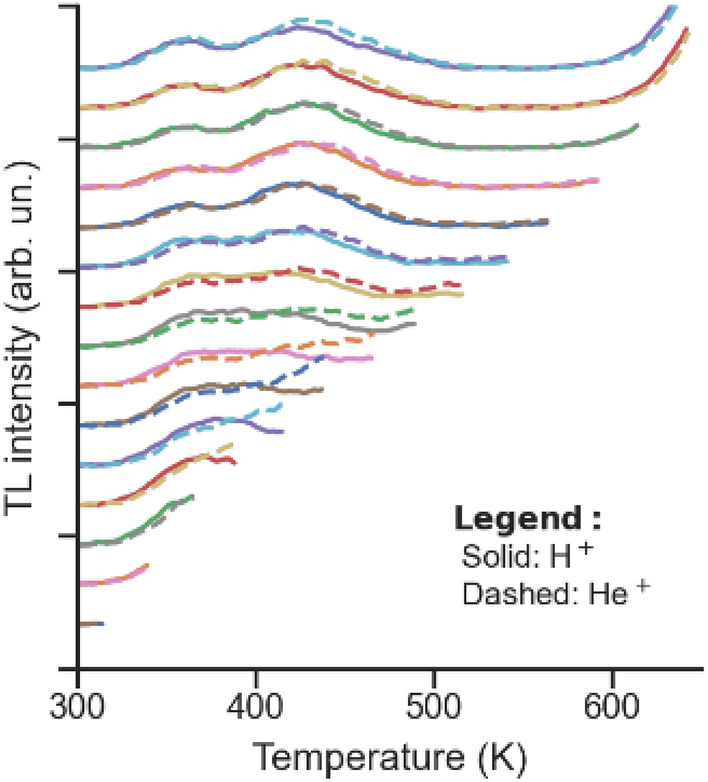
TL glow curves obtained with an incremental final temperature and for H^+^ irradiation (solid lines) and He^+^ (dashed lines). The curves were vertically shifted for clarity. These glow curves were not corrected for the background.

With the possibility of inducing different types of defects with different ions in mind, considering the IBIL results, a TL experiment was performed on a sample which was homogeneously irradiated with either 600 keV H^+^ or 2000 keV He^+^ up to fluences of 5.8 × 10^15^ H^+^/cm^2^ and 5.0 × 10^14^ He^+^/cm^2^ (i.e., in a ratio of ~ 11.5:1). Therefore, the vacancy profile according to SRIM simulations should be similar in both cases (see the supplementary material). Moreover, considering the difference between the first and the subsequent TL measurements, as shown in Fig. 3, the TL signal was acquired in several steps. After an initial 400 s *β*^*−*^ irradiation, in each measurement step, the temperature was raised from room temperature (~ 300 K) with a constant heating rate of 2 K/s, while the final temperature was gradually increased in steps of 25 K up to 650 K. Therefore, instead of raising the temperature directly to 650 K, this method made it possible to continuously monitor the evolution of the glow curves, which is important if the induced defects are removed over the course of a measurement. The glow curves are plotted in Fig. 4. It is important to mention that the glow curves shown are not normalized and were obtained in similar measurement conditions. The Cr-doped sample was first irradiated with H^+^ ions, measured by TL and then subjected to a rapid thermal annealing treatment at 1073 K for 2 min in an Ar atmosphere. This temperature is above those which were shown in the literature to be efficient in removing the irradiation-induced defects^30^, and also efficiently reverses the enhancement of the Cr^3+^ luminescence^23^. Afterwards, the sample was irradiated with He^+^ ions and measured again by TL. Hence, the irradiated volume and the vacancy profile were kept constant in both measurements, which were performed in the same heating and acquisition systems; thus the TL intensities are directly comparable.

It can then be noticed that for final temperatures *T*_STOP_ between 400 and 500 K, the glow curves obtained upon H^+^ and He^+^ irradiation diverge significantly; however, for *T*_STOP_ above 525 K, the curves almost coincide again. This observation may be explained by the induction of different kinds of defects in each of the irradiations. In particular, the curve obtained when heating up to 450 K suggests the presence of a glow peak above 450 K for the He^+^ irradiated sample, but such a structure is no longer observed at higher temperatures. Thus, this hints at the presence of a defect of a different nature, that was subsequently removed when the sample was heated up to a temperature of ~ 500 K. Hence, the TL results agree with the hypothesis formulated above, in the context of the IBIL results, regarding the nature of the defects induced by the H^+^ irradiation when compared with the He^+^ irradiation. However, once the sample is heated up to a temperature of ~ 500 K, the glow curves become very similar, being also very similar to the ones shown in Fig. 3. Therefore, this suggests that the defects present at the end of the measurement are similar in both cases. Moreover, this is also in agreement with previous studies where damage recovery was observed to take place at temperatures between ~ 450 and ~ 650 K^30^.

## Conclusions

In conclusion, this work shows an enhancement of the Cr^3+^ IBIL yield in Cr-doped *β*-Ga_2_O_3_, which is observed using both 600 keV H^+^ and 2000 keV He^+^ ions, with similar expected vacancy profiles; it thus shows that the luminescence activation does not depend on the incident ion itself, but rather on the irradiation-induced defects. On the other hand, the unexpectedly similar cross-sections for the conversion of pristine to radiative centers obtained for both ions suggest that the nature of the induced defects is different in each case, with H^+^ irradiation being more efficient in sensitizing the Cr^3+^ emission than the He^+^ irradiation, i.e. the ratio of the cross-section for conversion of pristine to radiative centers to the cross-section for conversion from radiative to non-radiative centers is higher for the case of H^+^ irradiation. Indeed, TL measurements performed to temperatures up to 500 K hint at the presence of an additional glow peak for the sample irradiated with He^+^ ions which is not present for the sample irradiated with H^+^ ions. Moreover, when measuring at temperatures above 500 K, the glow peak is not observed, which suggests that the defect associated with it is removed.

Subsequent TL measurements are in agreement with the IBIL results, showing that the ion irradiation affects the existent electron traps and/or the trapping/recombination dynamics. In particular, they reveal that the sample only displays a TL signal after the irradiation. This is consistent with the reported enhancement of the IBIL associated with the Cr^3+^ emission with the ion irradiation fluence in the sample. Finally, the TL signal associated with the Cr^3+^ ions is quenched upon annealing at 923 K, due to the removal of the defects responsible for the sensitization. This temperature agrees with previous studies, where a temperature of 650 K is shown to be efficient in removing proton-induced defects, as measured by DLTS^30^, and a temperature of 823 K was shown to be efficient in quenching the Cr^3+^ IBIL^23^.

This study therefore contributes to a better understanding of irradiation defects in *β*-Ga_2_O_3_ and how they can sensitize the Cr^3+^ luminescence. Moreover, it reveals that Cr-doped *β*-Ga_2_O_3_ crystals can be potentially explored as reusable optical sensors for ionizing radiation and dosimeters, working both in and ex situ.

See the supplementary material for more information about the performed SRIM simulations and more detailed TL measurements and results.

## Methods

### Materials

Single-crystal (100)-oriented, Cr-doped *β*-Ga_2_O_3_ was grown by a modified Edge-defined Film-fed Growth method at the State Key Laboratory of Crystal Materials ^36^.

### Ion-beam-induced-luminescence

IBIL was performed at the μ-probe set-up installed at the 2.5 MV Van de Graaff accelerator at the Laboratory of Accelerators and Radiation Technologies of Instituto Superior Técnico, Universidade de Lisboa^23,37^. The nominal beam current for the 600 keV H^+^ irradiation was 400 pA, with an irradiation area of 4.3 × 10^−3^ cm^2^. For the 2000 keV He^+^ irradiation, the nominal current was 100 pA with an irradiation area of 4.0 × 10^−3^ cm^2^.

### Thermoluminescence

TL was performed using a Risø Thermoluminescence/Optically-stimulated luminescence reader (TL/OSL-DA-20), manufactured by DTU Physics, at the Luminescence Dating Laboratory of Instituto Superior Técnico, Universidade de Lisboa. The excitation was performed using a ^90^Sr/^90^Y *β*^*−*^ source (with a nominal activity of 40 mCi and a dose rate in quartz of 0.073 ± 0.002 Gy/s). Each irradiation had a duration of 400 s and a heating rate of 2 K/s was employed. A commercial detection filter RG630 was employed in order to monitor the spectral region with wavelengths above (630 ± 6) nm, which coincides with the upper detection limit of 630 nm of the PMT.

## Supplementary Information

Supplementary Information.

## Data Availability

The data that support the findings of this study are available from the corresponding author upon reasonable request. The authors have no conflicts to disclose.
