# Supplementary material for: Probing the Cr3+ luminescence sensitization in β-Ga2O3 with ion-beam-induced luminescence and thermoluminescence
Source: Sci Rep. 2023 Mar 25;13:4882. doi: 10.1038/s41598-023-31824-0 (PMC10039926; doi:10.1038/s41598-023-31824-0)
Supplement: Supplementary file 1 — Supplementary Information. [file 41598_2023_31824_MOESM1_ESM.docx]

**Supplementary Material**

**Probing the Cr^3+^ luminescence sensitization in *β*-Ga_2_O_3_ with
ion-beam-induced luminescence and thermoluminescence**

D. M. Esteves^1,2^*, A. L. Rodrigues^3^, L. C. Alves^3,4^, E. Alves^2,4^, M. I. Dias^3,4^,
Z. Jia^5^, W. Mu^5^, K. Lorenz^1,2,4^, M. Peres^1,2,4^

*^1^ INESC MN, Rua Alves Redol 9, Lisboa 1000-029, Portugal*

*^2^ IPFN, Instituto Superior Técnico, University of Lisbon, Av. Rovisco Pais 1, Lisboa 1049-001, Portugal*

*^3^ C^2^TN, Instituto Superior Técnico, University of Lisbon, Estrada Nacional 10, km 139.7, Bobadela 2695-066, Portugal*

*^4^ DECN, Instituto Superior Técnico, University of Lisbon, Estrada Nacional 10, km 139.7, Bobadela 2695-066, Portugal*

*^5^ State Key Laboratory of Crystal Materials, Shandong University, Shandanan Street 27, Jinan 250100, China*

** Corresponding author — email:* duarte.esteves@tecnico.ulisboa.pt

I. Monte Carlo damage simulations

Fig. S1 shows *Stopping and Ranges of Ions in Matter* (SRIM) Monte Carlo simulations^1^ performed for 600 keV H^+^ and 2000 keV He^+^ ions in *β*-Ga_2_O_3_, with displacement energies of 25 eV and 28 eV for Ga and O, respectively. The energies are chosen so as to achieve similar vacancy profiles for the two ions, as shown in Fig. S1 a). The defect concentration in the case of H^+^ is smaller than that of He^+^ by a factor of ~11.5. Hence, to create the same number of vacancies in a given sample, it must be irradiated up to fluences ~11.5 times higher for H^+^ compared to He^+^, as also shown in Fig. S1 a). The ionizations caused by the beam particles are also very important in an ion-beam-induced luminescence (IBIL) measurement, since they are responsible for generating the electron-hole pairs that subsequently recombine radiatively at the Cr^3+^ ions. These ionization profiles are shown in Fig. S1 b) for the two ions. The ionization is about four times higher for He^+^ than for H^+^. Therefore, in order to achieve similar excitation densities, the beam current was chosen four times smaller for He^+^ than for H^+^. Although ionization processes may lead to the change of the charge state of defects, we assume that the changes observed during the IBIL measurements are due to lattice defect formation. This assumption is corroborated by the fact that electron irradiation during the thermoluminescence (TL) measurement (see the main paper) does not lead to any sensitization of Cr^3+^ emissions.

The irradiation for the TL measurements was performed using three different energies (1.0, 1.5 and 2.0 MeV) in order to achieve a large irradiated volume where most of the defects are created. The vacancy distribution as a function of the sample depth according to SRIM simulations is shown in Fig. S2, and indicate that the first ~25 μm are damaged.


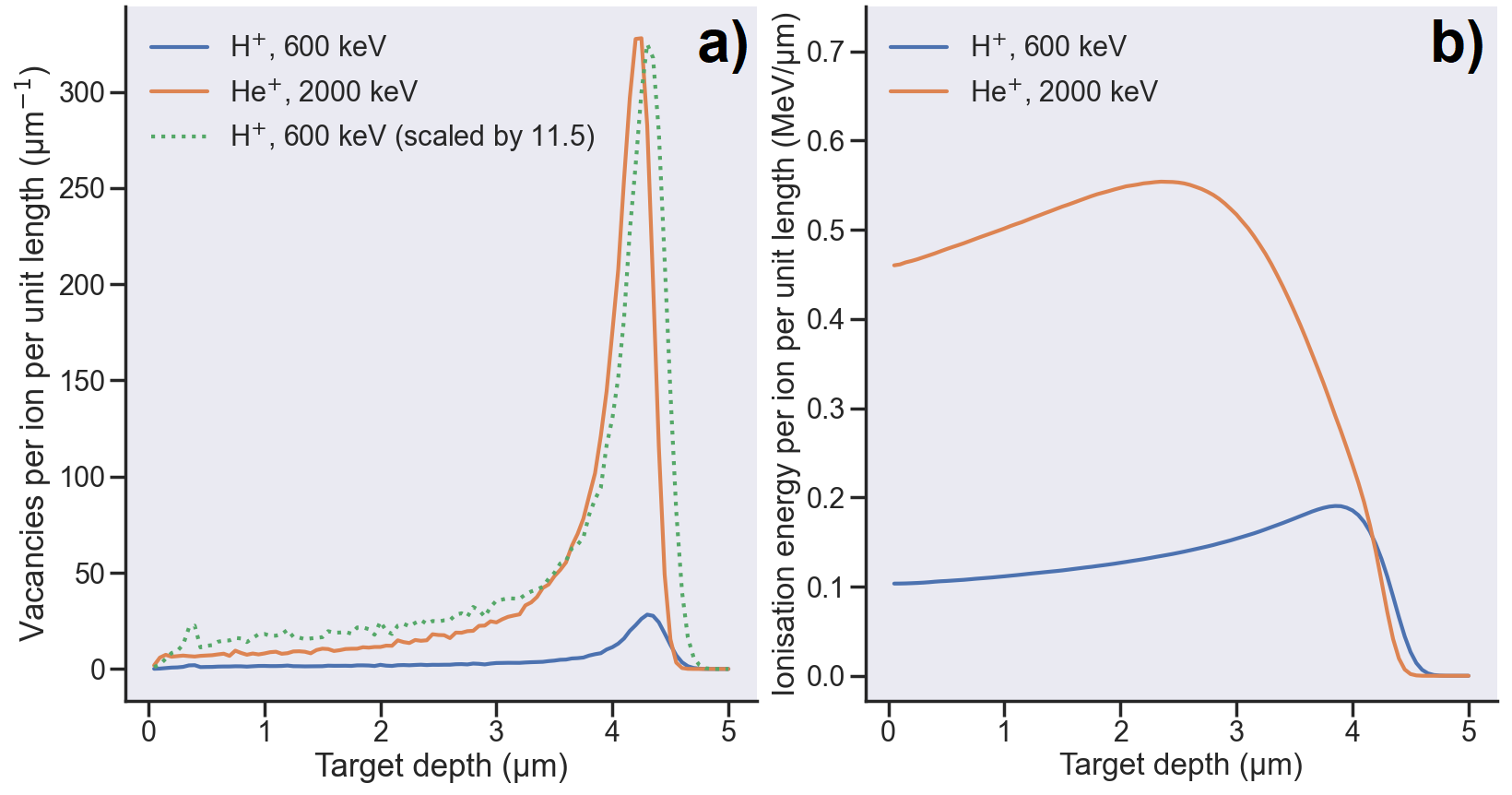


**Figure S1.** a) Number of vacancies per unit length and per incident ion, as simulated by SRIM, for 600 keV H^+^ and 2000 keV He^+^ ions. The H^+^ vacancy profile scaled by a factor 11.5 is similar to that of He^+^. b) Energy lost by ionization per unit length and per incident ion, as simulated by SRIM, for 600 keV H^+^ and 2000 keV He^+^ ions.


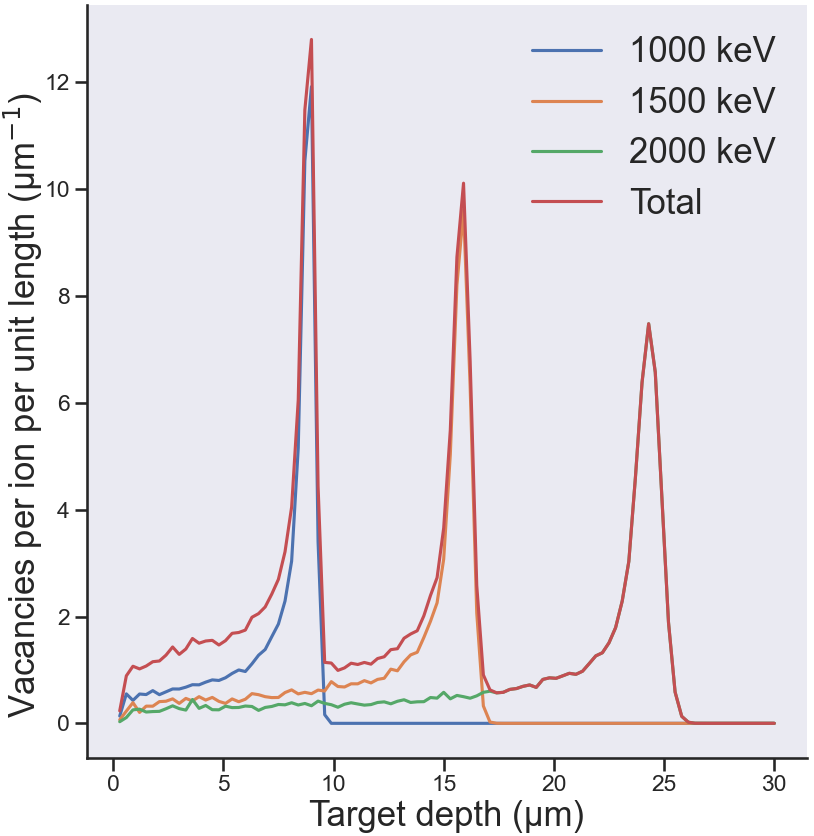


Figure S2. a) Number of vacancies per unit length and per incident ion, as simulated by SRIM, for 1.0, 1.5 and 2.0 MeV H^+^ ions, as well as the total final profile.

II. Thermoluminescence measurements using the *T*_M_–*T*_STOP_ method

The *T*_M_–*T*_STOP_ method consists in carrying out a preheating step before performing a full TL measurement^2^. Therefore, after each 400 s *β*^–^ irradiation, the sample was preheated to increasingly higher temperatures *T*_STOP_ with a 5 K step; after each preheat, a full TL measurement was performed up to 620 K, with a heating rate of 2 K/s. The position of the first maximum of the glow curve, *T*_M_, is then monitored as a function of the preheating temperature *T*_STOP_. This procedure allows glow peaks to be progressively removed, which helps in situations where peaks are overlapped and enables one to locate the position and the number of glow peaks with higher confidence. In particular, in a *T*_M_ vs. *T*_STOP_ plot, each glow peak can be assigned to a plateau (*i.e.*, when the value of *T*_M_ does not vary much with *T*_STOP_).

Additionally, this method also supplies some information regarding the trapping/detrapping kinetics, since it depends on the concentration of trapped electrons, which is progressively decreased^3^. In particular, discontinuous and abrupt steps correspond to first order kinetics, where the electron retrapping rate is negligible. On the other hand, a smoother transition between the steps corresponds to non-first order kinetics, such as second order kinetics, where the electron retrapping and electron-hole recombination probabilities are assumed to be equal^3^, or to an overlap of several first order peaks. The results are shown in Fig. S3.

The three clear horizontal portions shown in Fig. S3 b), indicating the position *T*_M_ of the first maximum of the glow curve for each preheat temperature *T*_STOP_, can be assigned to glow peaks. The discontinuous step observed from the plateau at 375 K to the one at 429 K is compatible with first order kinetics. On the other hand, the transition between the 429 K and the 498 K plateau is much smoother, which suggests non-first order kinetics^3^. Moreover, the almost-linear region between ~400 and ~475 K might correspond to a quasi-continuous distribution of closely-spaced energy levels^4^.


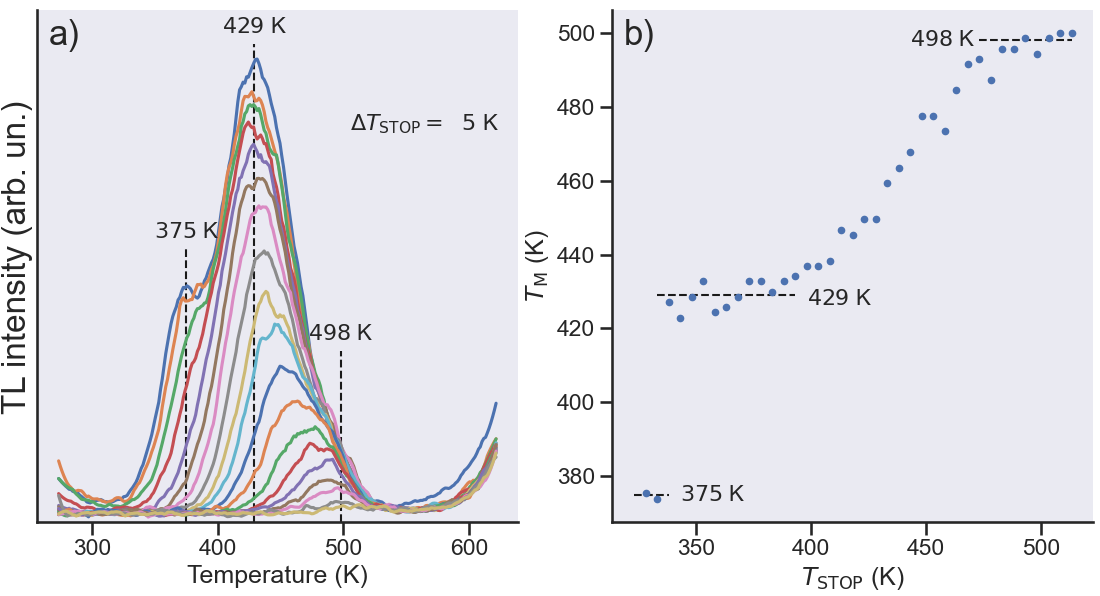


**Figure S3.** a) TL glow curves obtained after H^+^ irradiation, using the *T*_M_*–T*_STOP_ method, with steps of ∆*T*_STOP_ = 5 K (only every second curve shown). b) Position *T*_M_ of the first maximum of the glow curve for each preheat temperature *T*_STOP_. The dashed lines correspond to the flat portions of the graph, indicating a well-defined glow peak.

III. Glow curve deconvolution and kinetic parameter analysis

Taking the results of the *T*_M_*–T*_STOP_ method into account, the TL glow curve was computationally deconvoluted^5^ using three glow peaks: one first order glow peak (corresponding to the plateau at 375 K in Fig. S3 b)) and two general order glow peaks. The first order kinetics glow peak, also known as a Randall-Wilkins peak, is given by the expression^6,7^:

| $I(T)=\frac{n_{0}s}{\beta}\exp\left( -\frac{E}{kT} \right)\exp\left[ -\frac{s}{\beta}\int_{T_{0}}^{T} \exp\left( -\frac{E}{kT'} \right) dT' \right],$ | (1) |
| --- | --- |

where $n_{0}$ is the concentration of trapped electrons, $s$ is the attempt-to-escape frequency, $\beta$ is the heating ramp, $T_{0}$ is the initial temperature, and $E$ is the activation energy of the trap. In the case of a general kinetics order $b$, also known as a May-Partridge glow peak, the fitting expression is given by^8^:

| $I(T)=\frac{n_{0}s''}{\beta}\exp\left( -\frac{E}{kT} \right)\left[ 1-(1-b)\frac{s''}{\beta}\int_{T_{0}}^{T} \exp\left( -\frac{E}{kT'} \right) dT' \right]^{\frac{b}{1-b}},$ | (2) |
| --- | --- |

where $s^{''}=s^{'n_{0}^{b-1}},$ with $s'$ being a pre-exponential factor with dimensions of $\left[ \mathrm{Length} \right]^{3(b-1)}{[\mathrm{Time}]}^{-1}$. In the limit where $b\to1$, Equation (2) reduces to equation (1).

Moreover, the background was modelled according to^9^:

| $c_{1}+c_{2}\exp(c_{3}T),$ | (3) |
| --- | --- |

where $c_{1}$, $c_{2}$ and $c_{3}$ are fitting parameters, in order to account for the dark current of the photomultiplier tube and the blackbody radiation. The fitting parameters are shown in Table S1 for each of the peaks and the fits are shown in Fig. S4 for both the first and second measurements presented in Fig. 3 of the main text.


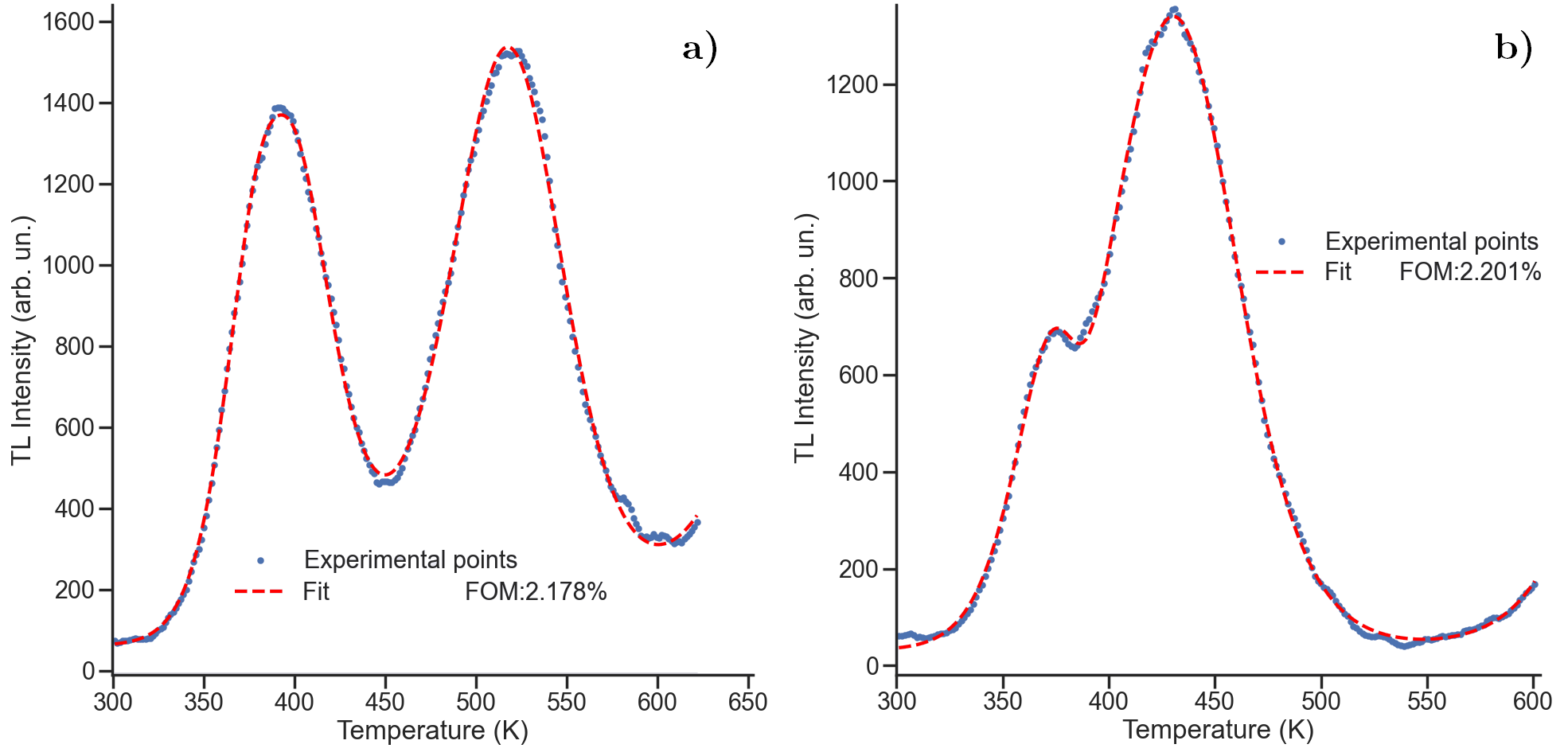


**Figure S4.** Fitting of the TL glow curve obtained on the first (a) and on the second measurements (b) of Fig. 3 in the main paper. This fit considers a first order glow peak (Equation (1)), two general order glow peaks (Equation (2)) and a background correction (Equation (3)). The figure of merit (FOM) is calculated with Equation (4).

| Glow peaks | Parameter | Values | |
| --- | --- | --- | --- |
|  |  | 1^st^ measurement | 2^nd^ measurement |
| $I$ | $s$ (Hz) | $2.92\times{10}^{10}$ | $1.68\times{10}^{10}$ |
|  | $b$ | $1$ | $1$ |
|  | $n_{0}$ (arb. un.) | $9.95\times{10}^{3}$ | $1.50\times{10}^{4}$ |
|  | $E$(eV) | $0.835$ | $0.812$ |
| $\mathrm{II}$ | $s''$ (Hz) | $1.41\times{10}^{9}$ | $6.74\times{10}^{7}$ |
|  | $b$ | $2.01$ | $1.76$ |
|  | $n_{0}$ (arb. un.) | $8.08\times{10}^{4}$ | $1.72\times{10}^{4}$ |
|  | $E$(eV) | $0.795$ | $0.718$ |
| $\mathrm{III}$ | $s''$ (Hz) | $3.39\times{10}^{7}$ | $5.75\times{10}^{6}$ |
|  | $b$ | $1.57$ | $1.59$ |
|  | $n_{0}$ (arb. un.) | $1.20\times{10}^{5}$ | $8.72\times{10}^{4}$ |
|  | $E$(eV) | $0.890$ | $0.677$ |
| Background | $c_{1}$ (arb. un.) | $61.4$ | $31.9$ |
|  | $c_{2}$ (arb. un.) | $9.76\times{10}^{-6}$ | $5.73\times{10}^{-11}$ |
|  | $c_{3}$ (K^-1^) | $0.0277$ | $0.0475$ |
|  | FOM | 2.178% | 2.201% |

**Table S1.** Fitting parameters of the fits shown in Fig. S4.

The differences in the estimated kinetic parameters are consistent with the removal of some defects when the sample is heated to temperatures above 600 K during the first TL measurement. In particular, while the first order peak (I) remained essentially unchanged, the activation energies pertaining to peaks II and III changed from about ~0.8 eV to about ~0.7 eV. Moreover, a large number of different traps have been identified in this energy range in several previous works in β-Ga_2_O_3_ under different doping conditions, by either TL or deep level transient/optical spectroscopy (DLTS/DLOS). Table S2 summarizes a few of these results, showing that the estimated energy levels are consistent with traps previously assigned to oxygen vacancies, complexes involving oxygen vacancies or Fe contaminants. In particular, it is consistent with the trap with an activation energy of 0.7 eV, previously correlated with the Cr^3+^ luminescence in the literature^10^. Moreover, no traps deeper than ~0.8 eV were observed, which may be related with the fact that the Fermi level for these electrically conductive samples lies close to the minimum of the conduction band.

| **Activation energy (eV)** | **Method** | **Doping** | **Assignment** | **Reference** |
| --- | --- | --- | --- | --- |
| 0.679 | TL | Fe | $V_{O}$-related defects | ^12^ |
| 0.694 | TL | Fe | $V_{O}$-related defects | ^12^ |
| 0.7 | TL | Mg, Mg+Cr | ${\mathrm{Cr}^{3+}-V}_{O}$ complex | ^10^ |
| 0.7 | TL | Mg | — | ^13^ |
| 0.722 | TL | Fe | Intrinsic defects | ^12^ |
| 0.75 | DLTS | Undoped | $Ga-O$ antisite or $V_{\mathrm{Ga}}-\mathrm{Ga}_{i}$ complex | ^14^ |
| 0.751 | TL | Undoped | $V_{O}$-related defects | ^12^ |
| 0.78 | TL | Undoped, Cr, Mg+Cr | Fe | ^10^ |
| 0.78 | DLTS | Undoped | Fe | ^14^ |
| 0.81 | DLTS/DLOS | Undoped | Extrinsic impurities (e.g. Fe or C) | ^15^ |
| 0.82 | DLTS/DLOS | Undoped | Sn or $V_{O}$ | ^16^ |
| 0.82 | DLTS/DLOS | Undoped | — | ^17^ |
| 0.84 | TL | Fe | Fe | ^18^ |
| 0.84 | TL | Mg | $V_{O}$ | ^13^ |

**Table S2.** Summary of different traps reported in the literature, obtained by TL or DLTS/DLOS measurements in β-Ga_2_O_3._

In thermoluminescence, the fitting quality is commonly assessed based on a figure of merit (FOM) defined as:

| $\mathrm{FOM}=\frac{\sum_{T} \left\vert I_{\exp}\left( T \right)-I_{\mathrm{fit}}(T) \right\vert}{\sum_{T} I_{\exp}(T)}\times100\%,$ | (4) |
| --- | --- |

where the sums are performed over the probed temperatures, $I_{\exp}$ refers to the experimentally measured TL intensity and $I_{\mathrm{fit}}$ refers to the fitted intensity. In the present case, the FOMs are below the commonly used threshold value of $2.5\%$^11^, thus indicating a good agreement between the fit and the experimental data. However, it should be mentioned that the deconvolution process is ambiguous and depends on the number of peaks (which was chosen here based on the results of the *T*_M_*–T*_STOP_ method) and on the initial guesses. Hence, other sets of parameters may also be able to accurately describe the glow curves.

IV. Irradiation with different ions

Fig. 4 of the main text shows the results for a TL experiment performed on a sample which was homogeneously irradiated with either 600 keV H^+^ or 2000 keV He^+^ to fluences of 5.8 × 10^15^ H^+^/cm^2^ and 5.0 × 10^14^ He^+^/cm^2^ (i.e., in a ratio of ~11.5:1), respectively. This ratio allows the vacancy profiles shown in Fig. S1 to be similar in both cases. Fig. S4 shows the same data (only every other curve is shown, for visual clarity) separated by the type of ion used in the irradiation. This figure clearly shows that for lower end temperatures the glow curves for the He^+^ irradiation are more intense than those of the H^+^ irradiation. However, for temperatures above ~500 K become very similar, thus suggesting that there are different traps involved with each irradiation condition, but the associated defect is removed for a heating temperature of 500 K.


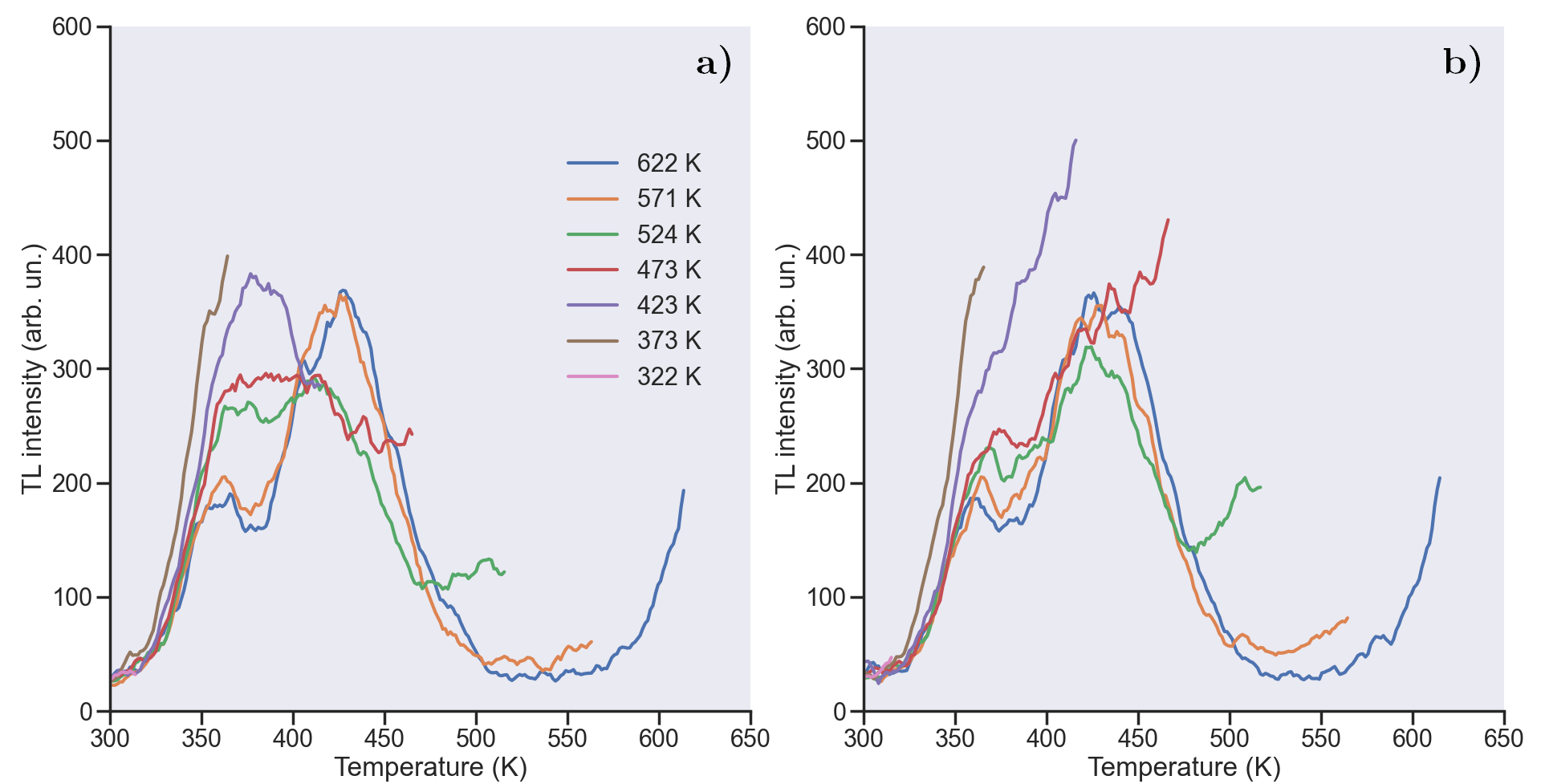


**Figure S5.** TL glow curves obtained with an incremental final temperature, obtained for H^+^ (a) and He^+^ (b) irradiations, respectively, as shown in Fig. 4 in the main paper.

**References**

^1^ J.F. Ziegler, J.P. Biersack, and U. Littmark, *SRIM – The Stopping and Range of Ions in Solids* (Pergamon, New York, 1985).

^2^ S.W.S. McKeever, Physica Status Solidi (a) **62**, 331 (1980).

^3^ S.W.S. McKeever, *Thermoluminescence of Solids* (Cambridge University Press, 1985).

^4^ O.Q. de Clercq, J. Du, P.F. Smet, J.J. Joos, and D. Poelman, Phys. Chem. Chem. Phys. **20**, 30455 (2018).

^5^ J.M. Kalita and M.L. Chithambo, J Lumin **185**, 72 (2017).

^6^ J. ~T. Randall and M. ~H. ~F. Wilkins, Proceedings of the Royal Society of London Series A **184**, 365 (1945).

^7^ J.T. Randall, M.H.F. Wilkins, and M.L.E. Oliphant, Proc R Soc Lond A Math Phys Sci **184**, 390 (1945).

^8^ C.E. May and J.A. Partridge, J Chem Phys **40**, 1401 (1964).

^9^ M. Puchalska and P. Bilski, Radiat Meas **41**, 659 (2006).

^10^ A. Luchechko, V. Vasyltsiv, L. Kostyk, O. Tsvetkova, and B. Pavlyk, ECS Journal of Solid State Science and Technology **9**, 45008 (2020).

^11^ H.G. Balian and N.W. Eddy, Nuclear Instruments and Methods **145**, 389 (1977).

^12^ M.M. Islam, D. Rana, A. Hernandez, M. Haseman, and F.A. Selim, J Appl Phys **125**, 55701 (2019).

^13^ A. Luchechko, V. Vasyltsiv, L. Kostyk, O. Tsvetkova, and A.I. Popov, Nucl Instrum Methods Phys Res B **441**, 12 (2019).

^14^ M.E. Ingebrigtsen, A.Yu. Kuznetsov, B.G. Svensson, G. Alfieri, A. Mihaila, U. Badstübner, A. Perron, L. Vines, and J.B. Varley, APL Mater **7**, 22510 (2019).

^15^ E. Farzana, M.F. Chaiken, T.E. Blue, A.R. Arehart, and S.A. Ringel, APL Mater **7**, 22502 (2019).

^16^ Z. Zhang, E. Farzana, A.R. Arehart, and S.A. Ringel, Appl Phys Lett **108**, 52105 (2016).

^17^ H. Gao, S. Muralidharan, N. Pronin, M.R. Karim, S.M. White, T. Asel, G. Foster, S. Krishnamoorthy, S. Rajan, L.R. Cao, M. Higashiwaki, H. von Wenckstern, M. Grundmann, H. Zhao, D.C. Look, and L.J. Brillson, Appl Phys Lett **112**, 242102 (2018).

^18^ C.A. Lenyk, T.D. Gustafson, L.E. Halliburton, and N.C. Giles, J Appl Phys **126**, 245701 (2019).
